# Glycaemic profile of children undergoing anaesthesia (GLYCANA) at Mercy James Centre in Malawi: an observational study

**DOI:** 10.1186/s12871-023-02073-5

**Published:** 2023-04-10

**Authors:** Furaha Nzanzu Blaise Pascal, Singatiya Stella Chikumbanje, Rachel Mbweza, Andrew Kumitawa, Tiyamike Kapalamula, Emma Thomson, Eric Borgstein, Gregor Pollach, Felix Namboya

**Affiliations:** 1 Mercy James Centre for Paediatric Surgery and Intensive Care, Queen Elizabeth Central Hospital, Blantyre, Malawi; 2 Faculty of Medicine, Université Catholique du Graben de Butembo, Butembo, Democratic Republic of Congo; 3 Department of Anaesthesia, Queen Elizabeth Central Hospital, Blantyre, Malawi; 4 Department of Anaesthesia and Intensive Care, Kamuzu University of Health Sciences, Blantyre, Malawi; 5 Department of Public Health, Kamuzu University of Health Sciences, Blantyre, Malawi; 6 Department of Surgery, Kamuzu University of Health Sciences, Blantyre, Malawi

**Keywords:** Glycaemia, Blood glucose, Hypoglycaemia, Hyperglycaemia, Mercy James Centre

## Abstract

**Background:**

Hypoglycaemia and hyperglycaemia may develop during anaesthesia and surgery in children and can lead to severe adverse clinical outcomes. No study, as far as we know, has investigated glucose homeostasis in children undergoing surgery in Malawi. The aim of this study was to assess perioperative glucose levels of the children undergoing anaesthesia at Mercy James Centre (MJC) for Paediatric Surgery, Blantyre, Malawi.

**Methodology:**

This was an observational cross-sectional study. We looked at 100 children aged 1 day to 15 years anaesthetised at MJC. Data were analysed using SPSS 28. Student t test and Analysis of the variance (ANOVA) were used to compare means. The level of significance was 5%.

**Results:**

Male children represented 68%. The median age was 2.2 years. Sixten percents of patient were underweight. Fasting times were prolonged for 87%. Maintenance IV fluid with 2.5% dextrose was given to 14%. Overall, there was a significant increase of glycaemia from induction of anaesthesia to the end of the procedure. Hypoglycaemia was rare. The mean fasting glycaemia was 99.04 mg/dL ± 1.8, 116.95 mg/dL ± 34.2 at 30 min into the procedure and 127.62 mg/dL ± 46.8 at the end of the procedure. The differences in means were statistically significant (p < 0.001). Prolonged fasting times was associated with lower blood glucose means whereas nutrition status, type of the procedure, addition of dextrose in the fluid, and duration of procedure were associated with higher glycaemia means.

**Conclusion:**

Glycaemia increases under anaesthesia and surgery. Recommended fasting times, optimising nutritional status, when possible, no dextrose or lower than 2.5% dextrose in IV maintenance fluid are possible strategies to maintain blood sugar homeostasis during paediatric surgery and anaesthesia.

## Background

Hypoglycaemia and hyperglycaemia may develop during anaesthesia and surgery in children and can lead to severe adverse clinical outcomes [1–3]. Hypoglycaemia is defined as a plasma glucose concentration less than 40 mg/dL to 50 mg/dL, regardless of age [4]. It often develops during fasting in children and mostly in neonates for they have limited glucose stores, high glucose demand and defective glucose production [4, 5]. It is also accentuated in children with sepsis and in neonates with asphyxia and hypothermia. Hypoglycaemia has been associated with neurodevelopmental problems and poor postoperative [2, 4, 6].

Pollach et al. assessed fasting times at Queen Elizabeth Hospital in Malawi and found that the recommended fasting times were not adhered to and were mostly prolonged [7]. Dennardt et al., studying the impact of fasting times on glucose, concluded that prolonged fasting times led to metabolic changes including ketoacidosis and low normal blood glucose concentrations in their institution. The changes in practice carried out in the same institution following the first study improved the outcomes [8, 9].

The stress of surgery and anaesthesia induces hyperglycaemia and insulin resistance [1, 2]. Hyperglycaemia increases the risk of hypoxic-ischaemic brain or spinal cord damage. It can also induce diuresis and consequently dehydration and electrolyte disturbances, especially in small preterm infants with immature tubular function. Hyperglycaemia increases perioperative morbidity and mortality [10].

Several strategies are used during the perioperative period to avoid hypo or hyperglycaemia in children. These include limiting the preoperative fasting times and administering intravenous (IV) glucose containing fluids perioperatively [1, 4, 11–13]. The practice of IV fluid and glucose administration varies from institution to institution. Several guidelines recommend glucose of concentrations between 1% and 2.5% to maintain glucose homeostasis [5, 11, 14].

Mercy James Centre (MJC) is a dedicated Paediatric Surgery and Intensive Care Centre in Malawi. At MJC, current anaesthesia practice is based on American Society of Anesthesiologists guidelines and other evidence-based practices in the field. Concretely, children are allowed to drink clear fluid up to 1 hour (h) before surgery. Intraoperatively, even though crystalloid fluids (Ringer’s lactate or normal saline) containing 2.5% dextrose for IV maintenance have been advocated, the choice of the dextrose concentration is left to the anaesthesia practitioner. This results in use of different glucose concentrations by different anaesthetists. Furthermore, blood glucose levels are not routinely checked perioperatively. This may lead to missing children with deranged blood glycaemia and exposing them to the risk of hypoglycaemia or hyperglycaemia and related complications.

No study, to our knowledge, has investigated glucose homeostasis in children undergoing anaesthesia in Malawi yet. There is a need to provide data to inform and improve existing protocols. The aim of this study was to assess perioperative blood glucose levels and variations of the children undergoing surgery and anaesthesia at MJC following current anaesthesia protocols and to determine factors associated with these variations.

## Methods

The main aim of this observational cross-sectional study was to assess the perioperative blood glucose levels of children aged from 1 day to 15 years undergoing anaesthesia at the MJC. The primary objective was to determine glycaemia variations perioperatively. Secondarily, we wanted to identify the factors associated with these variations and to determine the incidence of hypoglycaemia and hyperglycaemia. The study was conducted between the 23rd of November 2021 and the 8th of January 2022.

The population of this study was constituted of an average of 135 children undergoing anaesthesia at the MJC per month. The sample size (n) was calculated using an Online Sample Calculator Tool from Select Statistical Services with finite population correction. The formula used was n = N*X / (X + N – 1) where X = Z_α/2_^2^ *p*(1-p) / MOE^2^ and Z_α/2_ is the critical value of the Normal distribution at α/2. At confidence level of 95%, α is 0.05 and the critical value is 1.96. MOE is the margin of error, p is the sample proportion which was 50% for our study since the actual proportion was unknown, and N (135) is the population size [15]. We recruited 100 children instead of 101 due to recruitment constraints. The inclusion criteria were children aged from 1 day to 15 years undergoing anaesthesia and whose parents or legal guardian were willing to give consent to participate in the study. The exclusion criteria were neonates less than 24 h old, children with diabetes mellitus, children coming for examination under anaesthesia and those whose operation was expected to last less than 30 minutes.

Data were collected using a data collection form designed for the study and from anaesthesia charts by trained data collecting clerks for consistency. The data collecting clerks observed the anaesthesia providers without interfering in their practice. For each patient, sociodemographic, nutritional, pre, intra, and postoperative information were collected. Sociodemographic and nutritional parameters included age, gender, weight, Mid-Upper-Arm Circumference (MUAC), and address. Preoperative variables included presence of prematurity, procedure planned, comorbidities, duration of starvation, fasting glycaemia, urgency of the procedure, and fluid given before surgery. Intraoperative and immediate postoperative variables included induction time, beginning of surgery time, type and dextrose concentration of intravenous (IV) maintenance fluid administered, intraoperative glycaemia, transfusion, end of anaesthesia and surgery times.

Age was stratified into 4 groups adapted from Job KM et al.: neonates from birth to 30 days, infants from 31 days to 2 years, children from 2.1 to 11 years and adolescents from above 11 years to 15 years [16]. Starvation time was noted to be prolonged if it exceeded 4 h for breastfeeding children and 6 h for children on a solid diet or formula feeds. We created a composite variable named nutritional status for each child. For children aged 0 day to 5 years, we calculated the weight-for-age z-scores according to gender, and for the children above 5 years we used MUAC to evaluate the nutritional status. The child was said to be malnourished/underweight if the weight-for-age z score was <-2 standard deviation (SD) or MUAC < 12.5 cm irrespective of gender and age. Above the z score + 2 SD irrespective of gender or MUAC above 21.8 cm for 5–9 years boys and 20.8 for girls or above 25.4 cm for 10–14 years boys and 24.8 for girls and above 27.8 cm for the 15 years irrespective of gender, the child was overweight [17, 18]. The remaining children were normal for nutritional status. The anaesthesia provider was either a non-physician anaesthesia clinical officer (ACO) or a physician anaesthesiologist.

For the type of surgery, medium laparotomy included stoma closures, pyloromyotomy and omphalocele closures. Major laparotomy included laparotomy for bowel obstruction, peritonitis, liver problem, lymphoma and relaparotomy after burst abdomen. Other surgery included chest drain placement, cutdown, mass excision and/or biopsy, laceration repair, skin graft, brachial fistula repair. Hirschsprung disease surgery (HD surgery) included transanal Soave’operation (TAS) and posterior sagittal anorectoplasty (PSARP).

The glycaemia was measured using capillary blood with an ACCU-CHECK® Instant glucometer made in Germany. In total 3 samples were taken from each participant. The first sample (S1) was collected as soon as the patient was induced before administration of any maintenance IV fluid. This was considered as the fasting blood sugar. The time of collection of this sample was recorded as time zero (T0). A second sample was collected 30 minutes (min) after the beginning of the surgery regardless of the beginning of the anaesthesia and was termed sample 2 (S2) at time 30 min (T30). A final sample (S3) was collected at the end of the operation (Time end or TE). If at any time of collection, the glycaemia was below or far above normal, the reading was noted, and the results were communicated to the anaesthesia provider to allow institution of appropriate management of the patient. The concentration of the glucose given to correct the hypoglycaemia was recorded. For this study, normal fasting values of glycaemia were 50-120 mg/dL for children aged 0–5 months and 70-100 mg/dL for 6 months old children and above [19].For all age groups, normal values were 70-150 mg/dL intra and postoperatively [14]. Hypoglycaemia and hyperglycaemia were defined as values below and above the normal glycaemia respectively.

Data were entered into an electronic database protected by a password using KoBoCollect App on a Smartphone. Each patient was given a unique identifier. No name was entered in the database. Data were then transferred into IBM SPSS 28 for analysis.

Frequencies were determined for categorial variables. For quantitative data, we calculated means or medians, range, and standard deviation according to the type of distribution. The differences between means were determined using T test, analysis of variance (ANOVA) and linear regression as needed. Associations between variables were established using Chi Square (χ^2^) test and p-values at level of significance of 5%.

## Results

### General characteristics

**Fig. 1:**
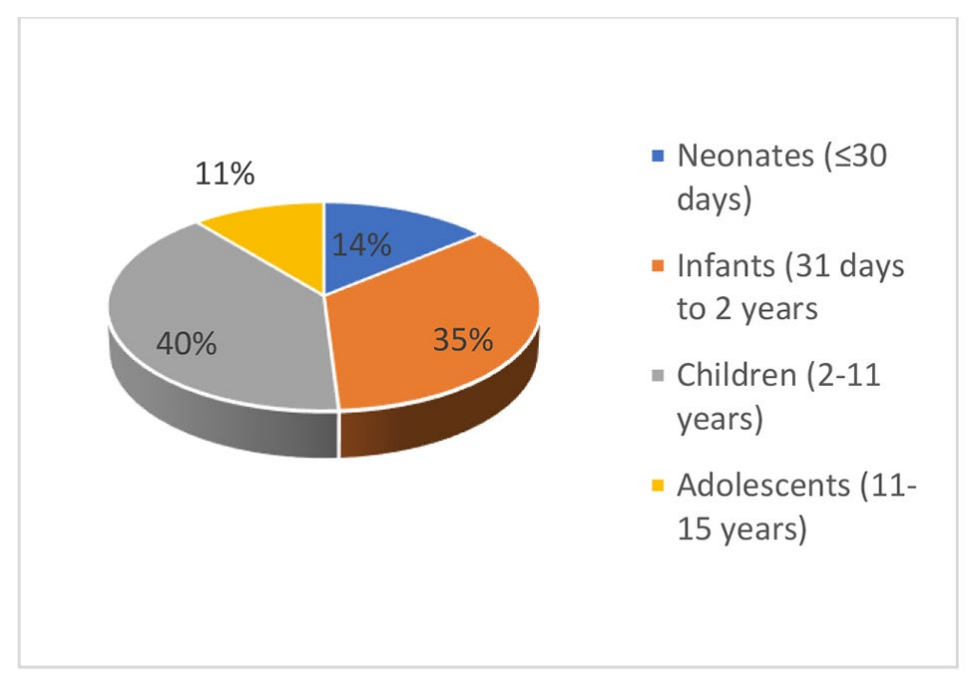
Age groups of the children

**Fig. 2:**
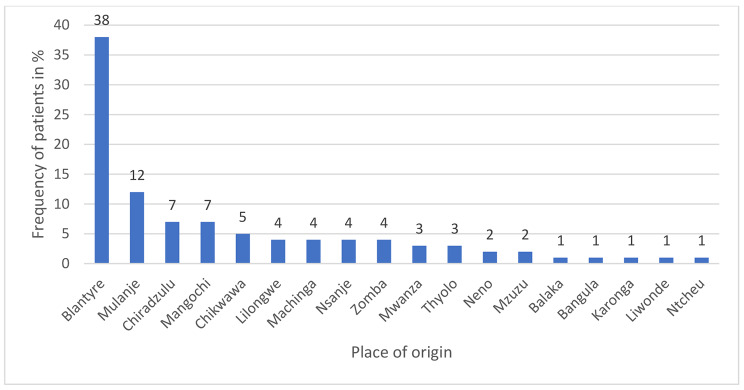
Origin of the children

**Fig. 3:**
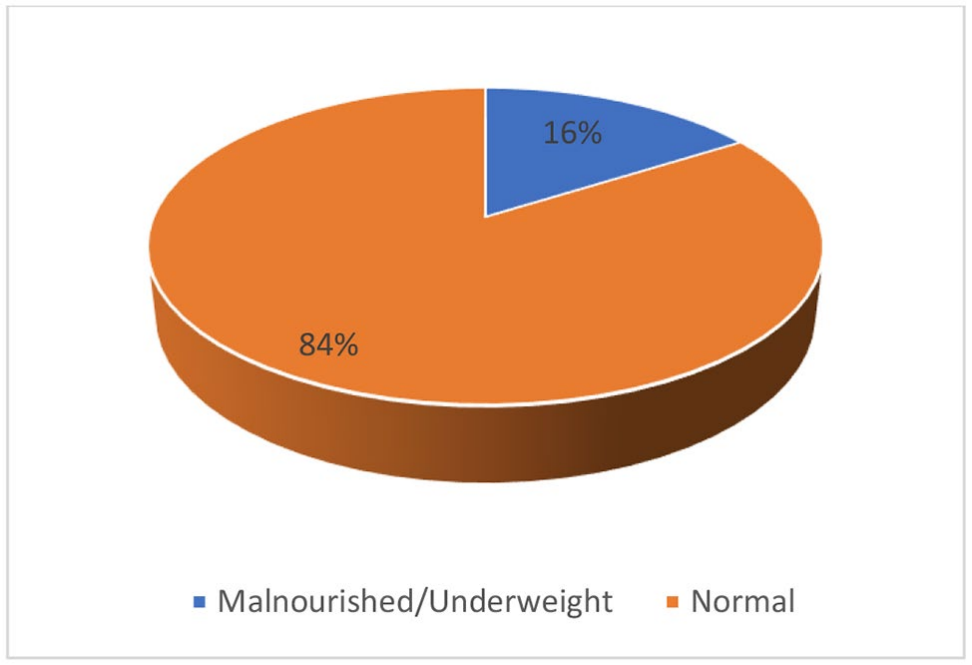
Nutrition status

**Fig. 4:**
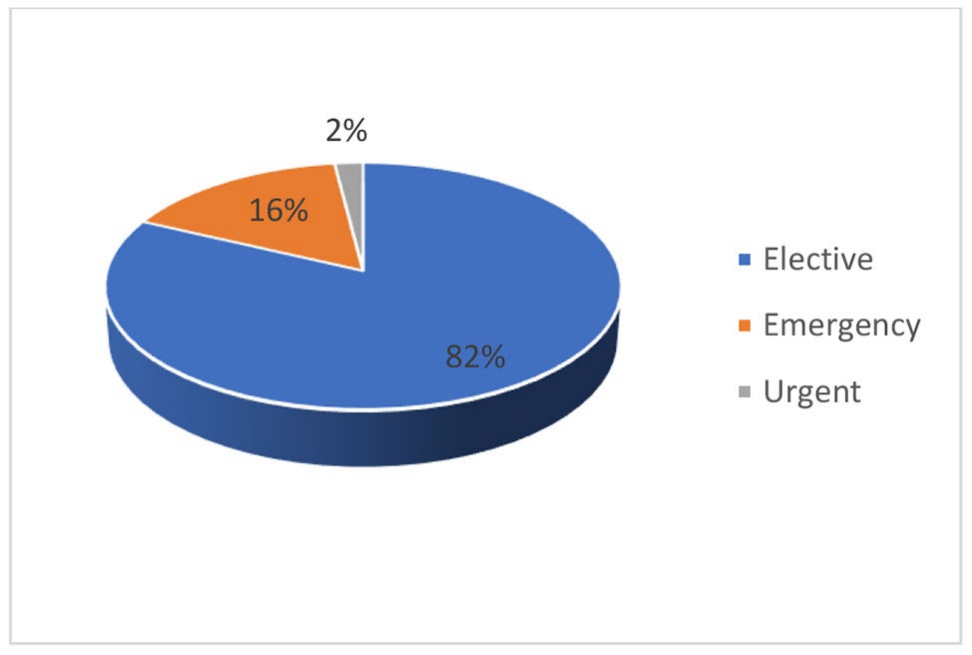
Urgency of the procedure

Male children represented 68% and female 32%. The median age was 2.2 years (minimum 2 days and maximum 15 years) (Fig. 1). Prematurity was found in 13% against 87% for full term. Mean weight was 14.67 ± 9.96 Kg. Most patients were referred from outside Blantyre (62%) (Fig. 2). Sixteen percents of the patients were malnourished/underweight (Fig. 3). Fasting times were prolonged for 87 patients (87%) and normal for 13%. The mean starvation time for breastfed children was 8 ± 4.3 h. For children taking solid food, the mean fasting time was 13.2 ± 3.7 h. Elective procedures represented 82% of the cases (Fig. 4). IV fluid preoperatively was given to 14% of the children with 6 patients (42.9%) receiving 10% dextrose, 5 (35.7%) plain crystalloid solutions and 3 (21.4%) 5% dextrose.

Anaesthesia clinical officers conducted 70% of the anaesthesia alone. All patients were done under general anaesthesia (GA) with different techniques for airway control. General anaesthesia with an endotracheal tube (GA ETT) represented 71% of all the GA. Maintenance fluid was Ringer’s lactate for 90%. Dextrose was added in the IV maintenance fluid for 18 (18%) patients with 14(14%) patients receiving 2.5% and 4 (4%) patients receiving 10% as a correction to hypoglycaemia episodes (Table 1). Transfusion was administered to 8 patients (8%) and 92% were not transfused. Mean Duration of anaesthesia and surgery were 127.17 ± 72.5 min and 88 ± 60.7 min.

**Table 1:** Intraoperative parameters

Intraoperative parameters	Frequency	Percentage
**Provider**		
ACO Alone	70	70.0
Anaesthesiologist + ACO	14	14.0
Anaesthesiologist alone	9	9.0
2 ACOs	7	7.0
**Type of anaesthesia**		
GA ETT	71	71.0
GA LMA*	24	24.0
GA MASK	3	3.0
GA TRACHEOSTOMY	2	2.0
**Type of maintenance IV Fluid**		
RL	90	90.0
NS	5	5.0
Both	5	5.0
**Maintenance IV Fluid Dextrose Concentration**		
Plain	82	82.0
Dextrose 2.5%	14	14.0
Dextrose 10%	4	4.0

*LMA Laryngeal mask Airway

### Perioperative glycaemia profile

The S1 mean glycaemia was 99.04 mg/dL ± 1.8. The S2 mean glycaemia was 116.95 mg/dL ± 34.2. The S3 mean glycaemia was 127.62 mg/dL ± 46.8. The ANOVA with repeated measurement revealed that these means were significantly different (p < 0.001).

Overall, there was a significant increase of glycaemia from S1 to S3. The increase difference from S1 to S3 was 28.6 mg/dL ± 39.7 with 95% CI of [20.7–36.5] with calculated t (t_c_) of 7.20, degrees of freedom (df) 99, and p-value < 0.001. The increase was also significant at 30 min. The difference of the mean between S1 and S2 glycaemia was 17.9 mg/dL ± 25.8 with 95% CI of [12.8–23.0], with t_c_ 6.938, df 99, and p-value < 0.001. The increase between S2 and S3 was 10.7 mg/dL ± 31.0 with 95% CI of [4.5–16.8], t_c_ 3.45, df 99, and p-value < 0.001.

Using ANOVA, age, gender, and weight, receiving fluid with additional dextrose preoperatively, receiving a transfusion were not associated with variations of the means of glycaemia at any time of the procedure (p-values > 0.05). The variation of S1 means were significantly associated with prolong fasting times. Patients with prolong fasting times had lower S1 (F 9.479, p = 0.003), S2 (F 4.335, p = 0.040) and S3 (F 5.441, p = 0.022) glycaemia means (Table 2). Nutritional status was significantly associated with glycaemia with malnourished/Underweight children presenting higher means of glycemia comparing to normal children for S1 (F 6.036, p < 0.016), S2 (F 8.113, p < 0.005) and S3 (F 5.239, p < 0.024) (Table 3).

**Fig. 5:**
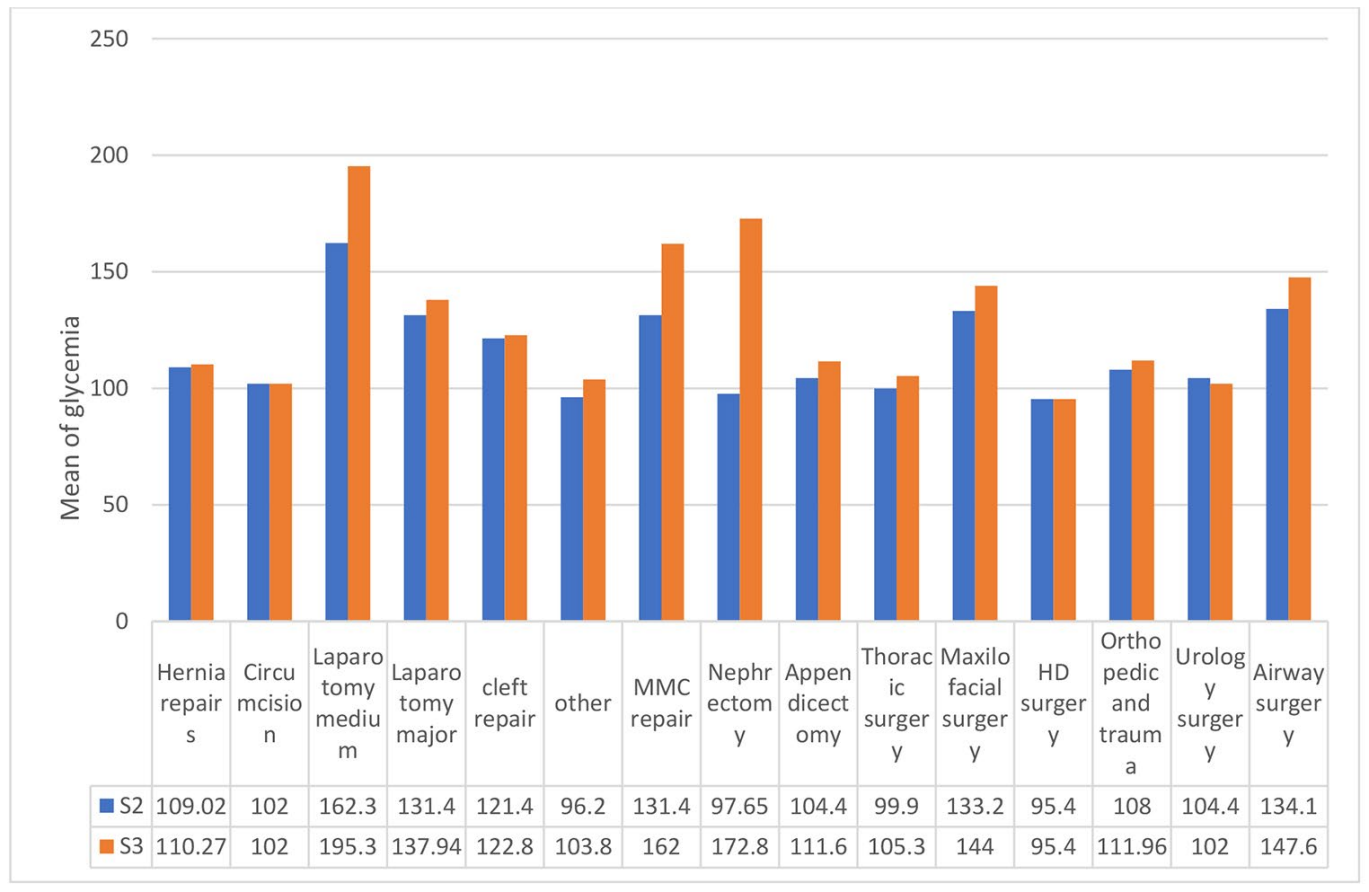
Means of Glycaemia according to type of surgery

During the operation, the differences were significantly associated with fasting times (Table 2), nutritional status (Table 3), type of the procedure, addition of dextrose in the fluid, concentration of dextrose employed, and duration of procedure, duration of the surgery. Malnourished children had 7.4 higher risk of developing hyperglycaemia at 30 min compared to normal children (OR 7.4 (CI 95% 2.17–25.21), χ^2^ 10.20, p < 0.001. The difference was not significant at the end of the procedure (χ^2^1.17, p = 0.27). After excluding patients who received 10% dextrose as correction for hypoglycaemia, patients who received 2.5% dextrose in IV fluid maintenance had significantly higher glycaemia means for S2 (F = 17.99, p < 0.001) and for S3 (F = 4.39, p = 0.03) (Table 4). Means varied significantly with duration of procedure (F = 3.50, p = 0.010) (Table 5). Mean glycaemia increased with time with a peak between 2 and 3 h. Medium laparotomies, airway surgery for S2 (F = 2.60, df = 14, p < 0.004) and medium laparotomies, nephrectomies and myelomeningoceles (MMC) surgery for S3 (F = 2.84, df = 14, p < 0.002) were associated with higher means (Fig. 5).

### Incidence of hypoglycaemia and hyperglycaemia

Glycaemia was normal for 63 patients (63%) at induction, 81 patients (81%) at 30 min into surgery and 77 patients (71%) at the end of the procedure. One patient (1%) was hypoglycaemic at induction, 4 patients (4%) were hypoglycaemic at 30 min from the beginning of the surgery, and 2 patients (2%) at the end of the procedure. We did not calculated associations for hypoglycaemia considering the low incidence of the event. For all hypoglycaemic patients, the hypoglycaemia was corrected soon after discovery with 10% glucose. Thirty-six patients (36%) had elevated fasting glycaemia at induction, 15 patients (15%) were hyperglycaemic at 30 min and 21 patients (21%) at the end of the procedure.

**Table 2:** Variations of means of glycemia according to fasting times

Variable	N	Mean	Std. Deviation	95% Confidence Interval for Mean		Minimum	Maximum
				Lower Bound	Upper Bound		
**Glycaemia at induction**							
**Fasting duration**							
Normal	13	114.65	22.209	101.23	128.07	74	149
Prolonged	87	96.70	19.206	92.61	100.80	59	160
Total	100	99.04	20.421	94.98	103.09	59	160
**Glycemia at 30 min**							
**Fasting duration**							
Normal	13	134.86	33.614	114.55	155.17	86	193
Prolonged	87	114.27	33.211	107.19	121.35	63	218
Total	100	116.95	33.816	110.24	123.66	63	218
**End procedure glycaemia**							
**Fasting duration**							
Normal	13	155.63	61.870	118.24	193.02	90	310
Prolonged	87	123.43	43.833	114.09	132.78	63	297
Total	100	127.62	47.449	118.21	137.03	63	310

**Table 3:** Variation of glycaemia according to nutritional status

	N	Mean	Std. Deviation	95% Confidence Interval for Mean		Minimum	Maximum
				Lower Bound	Upper Bound		
**Glycaemia at induction**							
**Nutritional status**							
Malnourished	16	110.25	25.170	96.84	123.66	59	153
Normal	84	96.90	18.816	92.82	100.98	61	160
Total	100	99.04	20.421	94.98	103.09	59	160
**Glycemia at 30 min**							
**Nutritional status**							
Malnourished	16	138.26	43.485	115.09	161.43	68	218
Normal	84	112.89	30.298	106.31	119.46	63	205
Total	100	116.95	33.816	110.24	123.66	63	218
**End procedure glycaemia**							
**Nutritional status**							
Malnourished	16	151.99	65.430	117.12	186.85	90	310
Normal	84	122.98	42.136	113.83	132.12	63	297
Total	100	127.62	47.449	118.21	137.03	63	310

**Table 4:** Variations of means of glycemia according of dextrose in the IV fluid

Parameters	N	Mean	Std. Deviation	95% Confidence Interval for Mean		Minimum	Maximum
				Lower Bound	Upper Bound		
**Glycemia at 30 min**							
**Dextrose %**							
0%	82	112.39	29.895	105.82	118.96	68	205
2.50%	14	150.04	35.319	129.65	170.44	76	218
**Total**	96	117.88	33.334	111.13	124.64	68	218
**End procedure glycaemia**							
**Dextrose %**							
0%	82	123.85	46.540	113.62	134.07	63	310
2.50%	14	151.71	42.464	127.20	176.23	76	243
**Totals**	96	127.91	46.811	118.43	137.40	63	310

**Table 5:** Variations of means of glycemia according to duration of the procedure

	N	Mean	Std. Deviation	95% Confidence Interval for Mean		Minimum	Maximum
				Lower Bound	Upper Bound		
**End procedure glycaemia**							
**Duration of procedure**							
Less than 60 min	18	105.00	24.865	92.63	117.37	63	162
61–120	40	120.19	32.009	109.96	130.43	74	221
121–180	21	155.06	66.914	124.60	185.52	63	310
181–240	13	129.32	41.594	104.19	154.46	83	223
More than 241 min	8	140.85	69.125	83.06	198.64	72	297
Total	100	127.62	47.449	118.21	137.03	63	310

## Discussion

This study assessed the glycaemia variations of paediatric patients undergoing anaesthesia and surgery at MJC, Malawi following current protocols. We found that overall blood glucose increased perioperatively however the variations depend on several factors. Age, gender, and weight were not associated with any variation of glycaemia perioperatively. These results corroborate those observed by Maitra et al. in India [20].

Fasting times were prolonged for most of the children. Prolonged fasting time was significantly associated with lower means of glycaemia at all the times perioperatively. All the children who were hypoglycaemic had prolonged fasting times. Pollach and his team observed similar prolonged fasting times which reduced with training of anaesthesia providers in 2014 in Malawi [7]. Similar results were observed in Nigeria and in Ethiopia [12, 21]. Fasting times are still a challenge in paediatric anaesthesia. Prolonged fasting times are harmful, cause discomfort, increase perioperative morbidity and are better avoided [5, 8, 9, 12, 20, 22]. These findings call for strategies to improve adherence to recommended fasting times to avoid adverse effects of prolonged fasting times. The strategies may include clear communication about fasting with parents and nurses on the ward, institution of individualised fasting time than the usual nil by mouth from midnight, refresher courses on fasting time for nurses in the wards and anaesthesia providers. Dennhardt et al. tried an optimised fasting time protocol in Germany which improved fasting times and perioperative morbidity [8].

ACOs performed 70% of all the anaesthesia procedures alone. In fact, there is still a shortage of physician anaesthetists in the country and the ACOs are the majority workforce available for anaesthesia provision as in many countries in the developing World. The country has only one paediatric anaesthesiologist. However, for safe anaesthesia, the World Health Organisation and the World Federation of Societies of Anaesthesiologist Anaesthesia Standards recommend a teamwork and the presence of a physician anaesthesiologist [23, 24].

Overall, there was an increase of glycaemia from induction of anaesthesia to the end of the procedure regardless of the type of fluid used for maintenance. The increase in glycaemia was more in malnourished children compared to normal children. These findings suggest that malnourished/underweight children overreact to surgical and anaesthesia stress compared to healthy children. Malnutrition has been found as a factor of increased catabolism during surgery and poor outcomes. Preoperative nutrition support (nutritional rehabilitation) may overcome these effects and improve outcomes [25, 26].

A higher percentage of children, mostly those who received additional dextrose in the maintenance fluids, were hyperglycaemic at the end of the procedure. These findings imply that hyperglycaemia is the greater problem during anaesthesia and surgery than hypoglycaemia which remained rare. Adding dextrose in the maintenance fluid exacerbated this known physiologic reaction to surgical and anaesthesia stress. Similar results were observed in the United States, Canada and in India [6, 14, 27, 28]. Hyperglycaemia is harmful during surgery and may increase perioperative morbidity and mortality. Hyperglycaemia is detrimental to the brain. It increases the risk of brain ischemia and anoxia and induces water and electrolytes disturbances. It may also cause postoperative bacteraemia in specific group of patients [1, 3, 5, 10, 11, 29–31]. In our centre, the current protocols suggest adding dextrose 2.5% to IV maintenance fluids. However, in this study, the few children who received additional dextrose in the IV maintenance fluids, were exposed to harmful higher glycaemia perioperatively. We thus suggest a revision of protocols towards lowering the dextrose to lower concentrations such as 1% or simply omitting routine additional dextrose to the fluid and instead to check glycaemia at the beginning and at least after 2 h into the procedure since this study demonstrated that the peak of glycaemia occurs around 2 to 3 h. Lower dextrose concentrations of 1% have been found to be effective in maintaining glycaemia homeostasis perioperatively [11, 14, 29, 32]. Moreover, there is a need to further investigate the outcomes of these children experiencing hyperglycaemia to further inform the protocols.

The longer the procedure the higher the glycaemia. In fact, long procedures are usually major ones and are associated with the highest surgical stress. This study also found that big procedures involving the abdomen (laparotomies) were associated with higher mean glycaemia. Surgical and anaesthesia stress form the basis of the pathophysiology of hyperglycaemia perioperatively [1, 3, 29, 33].

**Limitations**.

This study has some limits. It did not explore the factors associated with episodes of hypoglycaemia nor hyperglycaemia. However, it has the merit to have pointed out the factors related to lower and higher means of glycaemia during paediatric anaesthesia and surgery which constitute a baseline for further investigations and changes in current protocols.

## Conclusion

This study has helped to understand the homeostasis of glycaemia in our settings. Glycaemia increased under anaesthesia and surgery. Malnutrition, adding dextrose to IV fluid, laparotomies and prolonged surgery are the factors associated with increased glycaemia intraoperatively. Prolonged fasting times are associated with lower means blood glucose although hypoglycaemia is a rare phenomenon. Adherence to recommended fasting times, optimising nutritional status to normal whenever possible, reducing the concentrations of dextrose to lower than 2.5% or omitting dextrose in the IV maintenance fluid and checking perioperative blood glucose instead for an informed decision are possible strategies to maintain blood glucose homeostasis during paediatric anaesthesia and surgery.

## Data Availability

The datasets supporting the findings of this study are available from the corresponding author on reasonable request.

## Abbreviations

ACO: anaesthesia clinical officer
ANOVA: Analysis of the variance
ETT: Endotracheal tube
GA: General anaesthesia
HD: Hirschsprung disease
IV: Intravenous
LMA: Laryngeal Mask Airway
MJC: Mercy James Centre
MUAC: Mid-Upper-Arm Circumference.
